# New Professors in Harvard University

**Published:** 1847-05

**Authors:** 


*New Professors in Harvard University.*—We learn that the Corporation of Harvard University, since the resignation of Dr. Warren, have appointed three new professors, two of whom are to be attached to the Massachusetts Medical College in Boston, and one to the University at Cambridge. The new incumbents are Oliver W. Holmes, M. D., Professor of Anatomy and Physiology; John B. S. Jackson, M. D., Professor of Pathological Anotomy, and Curator; and Jeffries Wyman, M. D., Hersey Professor of Anatomy at Cambridge.—*Bost. Med. and Surg. Journ.*

